# Carbonic Anhydrase Inhibitors from Marine Natural Products

**DOI:** 10.3390/md20110721

**Published:** 2022-11-17

**Authors:** Claudiu T. Supuran

**Affiliations:** NEUROFARBA Department, Sezione di Scienze Farmaceutiche e Nutraceutiche, Università degli Studi di Firenze, Via Ugo Schiff 6, 50019 Firenze, Italy; claudiu.supuran@unifi.it

**Keywords:** carbonic anhydrase, natural product, sulfonamides, phenols, polyamines, coumarins, psammaplin C

## Abstract

Carbonic anhydrases (CAs, EC 4.2.1.1) are widespread metalloenzymes in organisms in all life kingdoms, being involved in pH regulation, metabolic processes and many other physiological and pathological conditions. CA inhibitors and activators thus possess applications as pharmacological agents in the management of a range of diseases. Marine natural products have allowed the identification of some highly interesting CA inhibitors, among which are sulfonamides, phenols, polyamines, coumarins and several other miscellaneous inhibitors, which are reviewed here. Psammaplin C and some bromophenols were the most investigated classes of such marine-based inhibitors and have been used as lead molecules for developing interesting types of potent and, in some cases, isoform-selective inhibitors, with applications as antitumor agents by inhibiting human CA XII and P-glycoprotein activities. Some phenols have shown interesting bacterial and fungal β-CA inhibitory effects. Marine natural products thus constitute a gold mine for identifying novel CA inhibitors, some of which may lead to the development of novel types of pharmacological agents.

## 1. Introduction

Carbon dioxide (CO_2_) is a colorless gas naturally existing in the atmosphere as a trace gas at about 0.04% (410 ppm), concentrations that tend to increase due to anthropic activities. CO_2_ is also a very stable form of carbon, the central element for life on Earth, with plants, algae and cyanobacteria using it for photosynthesis to produce carbohydrates from inorganic starting materials with light as an energy source. Oxygen is thus produced as a “side” product, which strongly influenced the evolution of life on Earth, since O_2_ is employed by aerobic organisms during their metabolism in life processes [1,2]. On the other hand, the reaction of CO_2_ with water leads to carbonic acid, an unstable compound that spontaneously splits into bicarbonate and a proton, but this reaction, which is essential for transforming a gas (CO_2_) into soluble products (bicarbonate and H^+^ ions), is very slow at physiological pH values [3,4,5]. Thus, catalysts evolved in all life kingdoms to efficiently catalyze this reaction, and these enzymes are known as carbonic anhydrases (CAs, EC 4.2.1.1) [3,4,5]. Eight genetically distinct CA families are known to date (α-ι-CAs), with a quite varied yet widespread distribution in prokaryotes and eukaryotes, with only very few bacteria and one mammal reported to not possess them to date [3,4,5,6]. Some of these enzymes are excellent catalysts for the conversion of CO_2_ into bicarbonate and protons, with some of them showing the highest turnover numbers among all known enzymes investigated to date, thus demonstrating their crucial role in the life cycle of organisms in which CAs are present [7,8]. Furthermore, because from two neutral molecules (CO_2_ and water), a weak base (bicarbonate) and a strong acid (H^+^ ions) are generated, CAs represent one of the most efficient and widespread systems for pH regulation in all biological systems [3,4,5,6]. However, these enzymes are not only pH regulators, as they are also involved (in addition to photosynthesis, already mentioned above [1,2]) in many other biochemical/physiological processes, such as metabolism (gluconeogenesis, fatty acid biosynthesis, de novo lipogenesis, urea biosynthesis, etc. [3,4,5,6,9]), electrolyte secretion and excretion [3,4,5,6,10], respiration [11], tumorigenesis [12], etc. Interfering with CA activity by using inhibitors and activators of these enzymes thus has profound physiological consequences, which have been exploited therapeutically for more than 6 decades for various pharmacological agents, such as diuretics, antiglaucoma, antiepileptic, antiobesity and antitumor agents [3,4,5,6,8,9,12]. Most CA inhibitors (CAIs) (and CA activators (CAAs)) that are in clinical use or are useful as pharmacological tools (see [3,4,5,6] for reviews in the field) are synthetic derivatives, which have been obtained over the years through classical drug design campaigns from synthetic lead compounds [4]. However, in the last decade, some natural products (NPs) have also started to be investigated for their CA inhibitory activity, which, in fact, led to significant advances in the field [13,14].

NPs represent a gold mine for identifying new chemotypes with biological activity [15,16] and new lead compounds [17,18,19,20], but ultimately, the genome mining and engineering of metabolic pathways have allowed the possibility of identifying totally new metabolic intermediates or highly diverse scaffolds for a range of pharmacological applications, mainly but not limited to anticancer and anti-infective agents [15,16]. Among the various NPs investigated so far, marine NPs constitute an important category of underexplored sources of chemical diversity, although in the last several years, there has been a renewed interest, and many advances in the field have been made [15,16,17,18,19,20].

In this article, I present marine NPs as a source of CAIs, a field that has not been reviewed until now. In an excellent article, Mujumdar and Poulsen [19] reviewed sulfonamide and sulfamate NPs as CAIs (however, the work is not entirely focused on marine NPs). Thus, as far as I know, this is the first exhaustive review in the field of marine-based CAIs in which all known classes of inhibitors are considered (sulfonamides, phenols, polyamines, coumarins, carboxylates, esters, etc.). As far as I know, no CAAs from marine NPs have been reported so far, but this type of modulator is currently less investigated [21].

## 2. Sulfonamides

Primary sulfonamides (RSO_2_NH_2_) incorporating aromatic, heterocyclic, aliphatic or sugar moieties represent the most common class of CAIs [4,22], with many effective (micro–subnanomolar) inhibitors reported to date against a multitude of enzymes from prokaryotes and eukaryotes (however, human CAs (hCAs), of which 15 isoforms are known to date, are the most investigated of such enzymes [3,4,5,6,21,22,23]). At least 25 clinically used drugs incorporating this group (and its isosteres, the sulfamate and sulfamide moieties) are in clinical use as diuretics and antiglaucoma, antiepileptic, and antiobesity agents [4,5], whereas many others are in clinical trials as antitumor agents [4,5,12] or anti-infectives [24]. Sulfonamides are zinc binders: through their deprotonated sulfonamide moiety, they bind the Zn(II) ion in the CA active site, participating in a multitude of other interactions with amino acid residues of the active site, which explains their efficient inhibition [4,13,23]. There are several hundred *X*-ray crystal structures of various sulfonamides bound to different CA isoforms in the literature reported to date [13,23].

### 2.1. Psammaplin C

Psammaplin C (compound **1**, Table 1) is one of the two primary sulfonamide NPs reported to date [19]. It was isolated from the marine sponge *Psammaplysilla purpurea*, later renamed *Pseudoceratina purpurea*, only in 1991 [25]. Psammaplin C incorporates a bromotyrosine-oxime-functionalized scaffold and an amide that links the bromophenol fragment to an ethylenesulfonamide moiety, leading to the presence of the primary sulfonamide group in this particular NP. The presence of this classical zinc-binding group (ZBG), associated, as mentioned above, with potent CA inhibitory action, prompted Poulsen and Supuran’s group to investigate the CA inhibitory properties of **1** [26].

Indeed, it has been observed that sulfonamide **1** effectively inhibits eight of the ten hCA isoforms investigated so far (Table 1), with inhibition constants in the range of 0.79 nM (against hCA XII, a tumor-associated isoform) to 379 nM (against hCA XIV) [26]. The two isoforms that are less sensitive to inhibition by this sulfonamide are hCA VI and XIII, for which micromolar K_I_s were measured (Table 1) [26]. It can also be observed that **1** has comparable or, in some cases, better CA inhibitory properties compared to the classical, standard sulfonamide CAI (acetazolamide, **AAZ**, 5-acteamido-1,3,4-thiadiazole-2-sulfonamide, a compound in clinical use [4]).

**Figure 1:**
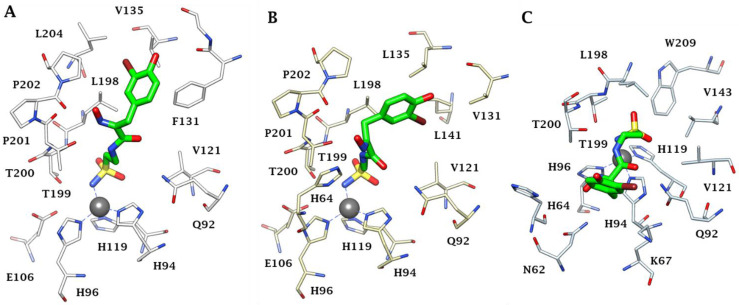
Active site view of (**A**) hCA II, (**B**) hCA IX mimic and (**C**) hCA XII mimic in adducts with Psammaplin C **1** (PDB codes 5A6H, 5G03 and 5G01, respectively). The zinc ion is shown as a gray sphere, and its three protein ligands (His94, 96 and 119) are displayed in CPK colors. The inhibitor is shown in green, whereas the residues involved in its binding are highlighted for all three CA isoforms (hCA I numbering system).

Furthermore, *X*-ray crystal structures of adducts of Psammaplin C **1** bound to hCA II (dominant cytosolic isoform [3,4]), an hCA IX (transmembrane, tumor-associated isoform [12]) mimic and an hCA XII (transmembrane, tumor-associated isoform [12,27]) mimic were also reported in the same work [26], rationalizing the effective (but diverse between different isoforms, see Table 1) inhibitory profile of the compound against these enzymes (Figure 1). Indeed, the deprotonated sulfonamide moiety of **1** is bound to the zinc ion and participates in the canonical interaction with the Thr199-Glu106 dyad, as in all CA–sulfonamide adducts reported so far [4,13,15,23]. Although the ethylenesulfonamide fragment of **1** is superimposable in the three adducts shown in Figure 1A–C, the amide and bromophenol fragments adopt very different orientations in the three adducts, making a large number of favorable but diverse interactions (H bonds, hydrophobic interactions and π-stacking) with numerous amino acid residues in the active sites of these enzymes, which are shown in detail in Figure 1A–C.

**Figure 2:**
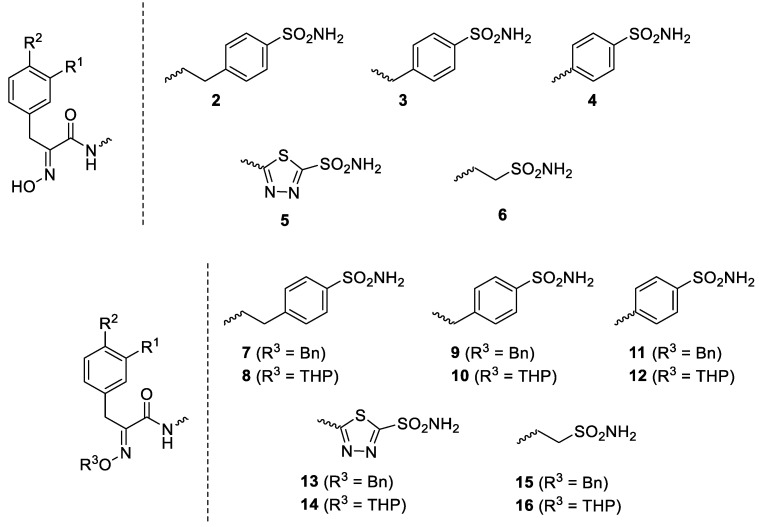
Synthetic Psammaplin C derivatives **2**–**16** [27]. Where not specified, R^1^ and R^2^ are H, Br and/or OH (interchangeable).

Considering the highly effective hCA XII inhibitory activity of **1** and the fact that this isoform is involved in tumorigenesis and resistance to antitumor drugs due to its pH-modulating and metabolic effects, as well as its action on P-glycoprotein (PgP) activity [27,28,29], a series of Psammaplin A derivatives of types **2**–**16** were reported and investigated as agents for overcoming temozolomide resistance in glioblastoma [27] (Figure 2).

The library of 45 Psammaplin C derivatives reported (compounds **2**–**16**) in the mentioned study [27] incorporated structural changes in all parts of lead compound **1**: (i) the bromotyrosine scaffold, with H, OH and Br groups interchanging with each other; (ii) the oxime part, which is kept free or protected by benzyl (Bn) or tetrahydropyran-2-yl (THP) groups; and (iii) the ethylene-sulfonamide fragment, which was changed to a benzenesulfonamide or 1,3,4-thiadiazole-2-sulfonamide fragment, with linkers with various distances to the amide moiety of the scaffold (Figure 2). Many of these structural changes afforded highly effective CAIs of the following isoforms: hCA I, II, IX and XII. For example, the acetazolamide-type Psammaplin C derivative (compound **5** with R^1^ = Br, R^2^ = OH) is a subnanomolar hCA XII and a low-nanomolar hCA IX inhibitor and was investigated in vitro and in vivo using samples from glioblastoma patients, showing enhanced activity in combination with the clinically used agent temozolomide [27]. This study [27] constitutes a very good example of how marine NPs such as Psammaplin C may lead to effective novel compounds that show significant antitumor activity.

### 2.2. Altemicidin

**Figure 3:**
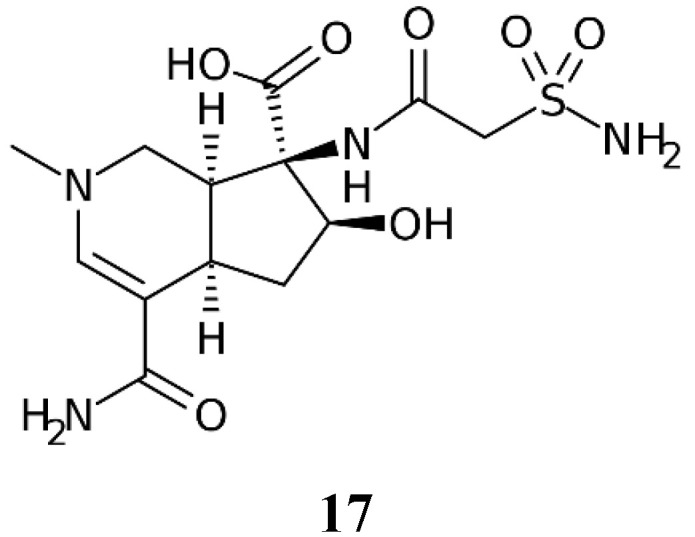
Chemical structure of Altemicidin **17**.

Altemicidin **17** (Figure 3) is the only other primary sulfonamide NP known so far [19].

The compound was isolated in 1989 from an actinomycete strain of marine origin, more specifically *Streptomyces sioyaensis* SA-1758, isolated in a sample of sea mud from Japan [30]. Being a primary sulfonamide with a carboxamido-sulfamoyl group connected to a bicyclic heterocyclic ring system, this compound probably shows potent CA inhibitory effects, which, however, have not been investigated to date, presumably due to the fact that the synthesis of this NP involves 27 synthetic steps [19], and the compound is highly expensive and difficult to find among chemical reagent providers. Hopefully, in the future, such investigations will be conducted, since the compound was originally reported to possess antitumor activity [30].

## 3. Phenols

Phenols were discovered to act as CAIs by Lindskog’s group in 1982 [31], whereas their inhibition mechanism was deciphered by Christianson’s group [32], who, working with simple phenol (PhOH) and hCA II, showed the inhibitor to be anchored to the zinc-coordinated water/hydroxide ion in the enzyme active site by means of H-bonding involving the phenolic OH moiety. In 1994, this was the first evidence [32] that CA inhibition mechanisms other than zinc binding may exist and thus furnished new classes of inhibitors, which, in fact, were thereafter discovered and explored in detail [23]. Many synthetic and NP-based phenols were in fact investigated as CAIs in the last several decades [33,34,35,36,37,38,39,40] after these seminal two findings and have been published [31,32]. Here, I will mention only marine NP-based phenol CAIs that have been investigated.

**Figure 4:**
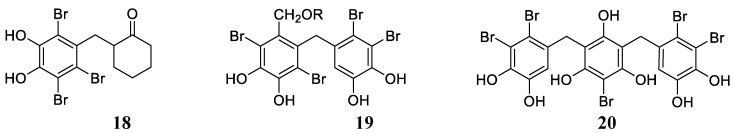
Examples of bromophenols **18**, **19** (R = H, Me, Et) and **20** (Vidalol B) isolated from marine organisms (red algae).

Bromophenols, either with one phenyl group (e.g., **18**, Figure 4 or more, as in **19** and **20** (Figure 4), and their derivatives are widespread compounds in many marine organisms, mainly red, brown and green algae, and they possess a range of biological activities, such as antimicrobial, antioxidant, anticancer, anti-thrombotic and anti-diabetic effects [17,18,41]. Balaydın et al. [42,43,44] were the first to investigate the CA inhibitory effects of some of these compounds as well as some of their synthetic analogs, working with isoforms hCA I, II, IV and VI.

**Figure 5:**
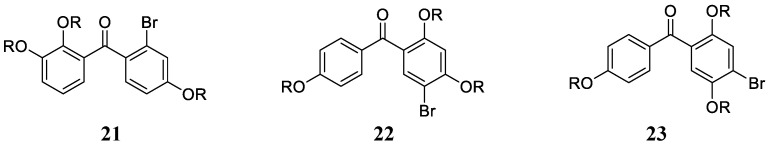
Bromophenols (R = H) and bromoethers (R = Me) investigated as CAIs by Taslimi et al. [45].

Derivatives of types **18** and **19** and some of their synthetic derivatives (i.e., ethers at the OH phenolic moieties) showed high micromolar inhibitory effects against hCA I, II, IV and VI [42,43]. Vidalol B **20**, 3,4,6-tribromo-5-(2,5-dibromo-3,4-dihydroxybenzyl)benzene-1,2-diol and its derivatives (e.g., 5,5′-methylenebis(3,4,6-tribromo-benzene-1,2-diols) were, in some cases, better inhibitors of the same CA isoforms, with a slightly selective profile and more effective inhibitory action against hCA IV and VI [44]. Inspired by the data obtained for marine NP bromophenols acting as CAIs [42,43,44], Taslimi et al. [45] reported a series of bromophenols and bromoethers **21**–**23** (Figure 5) incorporating a benzophenone scaffold, which showed enhanced inhibition against isoforms hCA I and II compared to the previously investigated derivatives, with K_I_s in the range of 2.6–6.0 µM against hCA I and 2.3–5.5 µM against hCA II.

**Figure 6:**
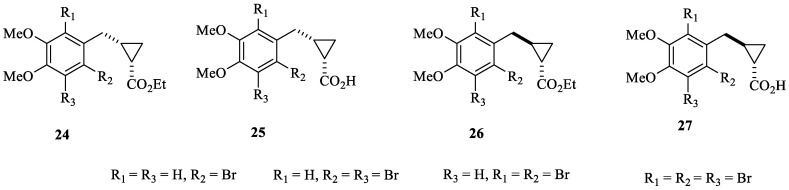
Dimethoxybromophenols incorporating cyclopropane moieties **24**–**27** investigated as hCA I, II, IX and XII inhibitors.

Probably the most interesting study that considered bromophenols as leads to obtain new CAIs was reported by Boztas et al. [46], who obtained a series of dimethoxybromophenol derivatives incorporating cyclopropane moieties of types **24**–**27** (Figure 6).

In addition to the cytosolic isoforms hCA I and II, dimethoxybromophenols **24**–**27** were also investigated as inhibitors of isoforms hCA IX and XII, involved, among others, in tumorigenesis [46]. Compounds **24**–**27** behaved as medium-potency inhibitors of all of these isoforms, with the most effective of them showing K_I_s < 10 µM. However, they did not show isoform-selective inhibitory profiles [46].

## 4. Polyamines

Polyamines such as spermine, spermidine and their derivatives were shown to act as CAIs by Carta et al. in 2010 [47]. Furthermore, by using stopped-flow kinetics and *X*-ray crystallography, it has been shown that these compounds, similar to phenols, do not act as zinc binders but anchor by means of their primary amino moiety to the water molecule/hydroxide ion coordinated to the zinc in the CA active site [47]. Although there are few other studies in which polyamines have been investigated as inhibitors of these enzymes, five natural product polyamines, **28**–**32** (Table 2), isolated from either marine sponges or fungi, were shown to act as inhibitors of six different hCA isozymes possessing therapeutic applications in drug development (Table 2) [48].

Polyamines **28**–**32** (which also incorporate phenol or bromophenol moieties, see above) are submicromolar hCA I, II and IX inhibitors and are more effective than spermine and have similar activity to spermidine [48]. Their activity is slightly lower for the inhibition of hCA IV, XII and XIV, but even against these isoforms, they act as efficient inhibitors. Unfortunately, there are no *X*-ray crystal structures that show which part of the molecule anchors to zinc-coordinated water (the amine or phenol one), nor have other synthetic compounds been obtained using **28**–**32** as leads.

## 5. Coumarins

**Figure 7:**
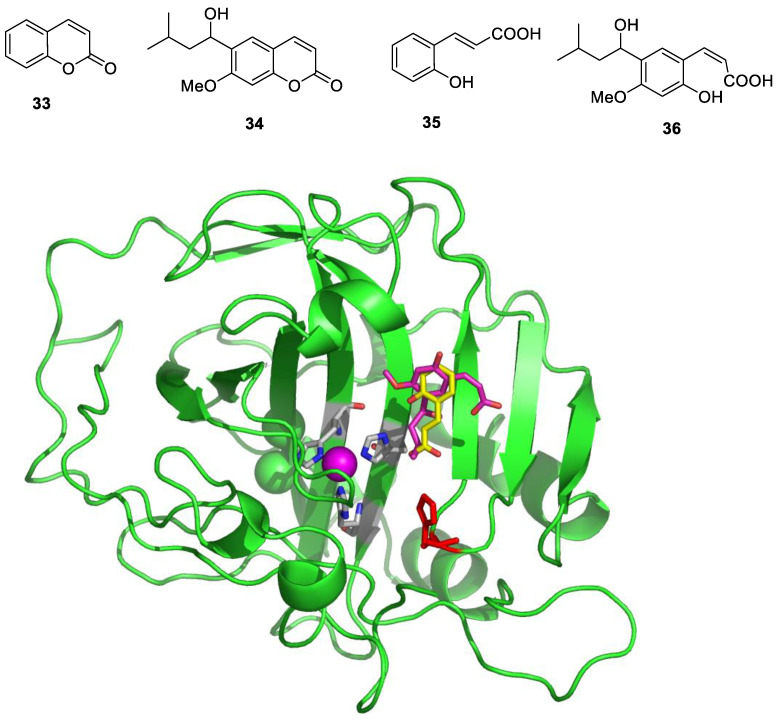
NP coumarins **33** and **34** and their hydrolysis products **35** and **36** bound to hCA II, shown in green, with the Zn(II) ion as a violet sphere and its three His ligands highlighted in gray. The two inhibitors are shown in yellow (**35**) and magenta (**36**), respectively, whereas the proton shuttle residue His64 is shown in red in two different orientations.

Coumarins are widespread NPs, being widely distributed in terrestrial and marine organisms [49,50]. They were first investigated as CAIs a decade ago, and it was demonstrated that both the simple derivative **33** as well as the NP derivative **34** (isolated from an Australian plant) [51] (Figure 7) act as effective CAIs but, more importantly, with a totally new inhibition mechanism [51,52].

Thus, coumarins indeed possess a unique CA inhibition mechanism, as determined from detailed kinetic, mass spectrometric and crystallographic experiments [51,52]. Unlike other CAIs discussed here, the formation of the enzyme–inhibitor complex is a slow process [51], indicating that coumarins act as suicide inhibitors, a hypothesis that was confirmed by *X*-ray crystallographic experiments in which hCA II and coumarins **33** and **34** were used [51,52]. Such data allowed researchers to observe that the coumarin ring undergoes hydrolysis via CA esterase activity, leading to the formation of 2-hydroxy-cinnamic acids **35** and **36**, bound at the entrance of the CA active site (Figure 7). It should be noted that 2-hydroxy-cinnamic acid **35** is bound to the enzyme in the *trans* geometry, whereas the bulkier derivative **36** was observed in the *cis* geometry, which is generally less stable (Figure 7). However, the most relevant finding was that the hydrolyzed coumarins bind at the entrance of the active site cavity at around 8–10 Å from the zinc ion, a region that is the most diverse in terms of its amino acid sequence among the 15 different human CA isoforms [3,4,5]. This may explain why coumarins and their derivatives are among the most isoform-selective CAIs reported to date [5,6,23].

Among the many NP coumarins investigated to date [50,53], some were isolated from marine organisms, and only these derivatives are discussed here.

Pentacyclic coumarins **37**–**39** were isolated from ascidians and were investigated as inhibitors of seven hCAs [53], as shown in Table 3. None of these compounds, similar to lead compounds **33** and **34** [51,52], inhibited the dominant and widespread isoform hCA II but showed low micromolar inhibition against several cytosolic (hCA I, VII and XIII) and trans-membrane (tumor-associated) isoforms, such as hCA IX and XII (Table 3).

## 6. Miscellaneous Inhibitors

Davis et al. [54] investigated a series of NPs, among which two derivatives isolated from ascidians, polyandrocarpamine A (**40**) and polyandrocarpamine B (**41**), were also identified as inhibitors of CAs from mammals (humans) and pathogenic bacteria and fungi, such as *Mycobaterium tuberculosis* and *Candida albicans* or *Cryptococcus neoformans* (Table 4).

Although possessing the phenol moiety, as in the compounds discussed earlier in this review, the two NPs **40** and **41** act as highly efficient inhibitors of the four β-CAs from pathogenic bacteria/fungi, with K_I_s in the submicromolar range, whereas they are quite ineffective as hCA I and II inhibitors (Table 4) [54]. It may be observed that the simple phenol derivative is much less effective as a β-CA inhibitor compared to **40** and **41**, which presumably indicates that it is not the phenolic OH moiety responsible for the inhibition but other moieties present in these NPs, such as the amidine or the imidazolidinone groups. Again, no *X*-ray crystal structures are available to allow a deep understanding of the CA inhibition mechanism with these interesting derivatives.

Rafiq et al. [55] recently reported that the methanolic extract of the marine alga *Dictyopteris hoytii* contains a multitude of NP compounds, among which are hydroxycinanmic acid derivatives, pentatetracontanoic acid, octadec-1-ene, epi-amyrine, terephthalaldehyde, tricosylic acid, hexadecanoic acid, lacceroic acid, methyl 2-bromobenzene 1,4-dicarboxylate, diethyl 2-bromobenzene 1,4-dicarboxylate, fucosterol, *n*-hexadecanoic acid, methyl ester, β-sitosterol, cerotic acid, *n*-octacos-9-enoic acid and 11-eicosenoic acid. These compounds were investigated as bovine CA II inhibitors [55], and high micromolar IC_50_ data were reported for most investigated derivatives. The compounds were docked within the hCA II active site, but from the presented figures, it is difficult to understand the quality of the computational work [55]. This preliminary work is indeed of interest, but the compounds should be investigated on more CA isoforms, and at least crystallization trials should be performed to elucidate their mechanism of inhibition.

## 7. Conclusions

The modulation of CA activity has multiple pharmacological applications, and although many CAIs have been in clinical use for decades [3,4,5,6], the search for more effective, less toxic and especially isoform-selective inhibitors (and also activators) continues, with many relevant discoveries being constantly reported [9,10,11,12,13,56]. In this context, the exploration of natural products of marine origin may add new relevant scaffolds and chemotypes to the already rich armamentarium of CAIs/CAAs. Indeed, over the last two decades, many such interesting compounds have been discovered in various marine organisms, including several classes of inhibitors, such as sulfonamides, phenols, polyamines, coumarins and carboxylates. For many of these derivatives, such as Psammaplin C, detailed synthetic, kinetic and crystallographic studies allowed a deep understanding of the inhibitory mechanism at the molecular level and fostered new drug design campaigns that led to even more efficient inhibitors, some of which showed relevant antitumor activity [26,27]. Phenols, especially bromo-substituted ones, have also constituted the subject of much research, and although various structural changes have been implemented, no highly effective, low-nanomolar compounds have yet emerged, nor have these compounds been crystallized in adducts with these enzymes, which is probably due to the rather high molecular weight induced by the presence of many bromine atoms in the investigated scaffolds [42,43,44,45,46,47,48,49]. Still, these compounds deserve more detailed investigations in order to better understand this class of CAIs. The same statement is valid for polyamines, as only five such marine NP compounds have been investigated to date.

Coumarins, on the other hand, have afforded some of the most isoform-selective CAIs reported to date [50,51,52,53], and their non-classical inhibition mechanism is understood at the molecular level, as several *X*-ray crystal structures are available [51,52]. However, more complex NP coumarins, such as those isolated from marine sources, have not been investigated in detail to date. Furthermore, more detailed pharmacological studies are needed with many such derivatives, as some of them indeed possess particularly interesting CA inhibition profiles.

There are also very few studies of non-mammalian CAs with marine NPs. The most detailed ones involve some ascidian phenolic derivatives (polyandrocarpamines) and also incorporate heterocyclic moieties [54], which probably do not exert their CA inhibition through the phenolic functionality. However, they are extremely effective inhibitors of pathogenic bacterial and fungal CAs from organisms (*Mycobaterium tuberculosis*, *Candida albicans* and *Cryptococcus neoformans*) known to have developed resistance to many anti-infective agents. Thus, detailed studies in this field may offer interesting new ideas for the development of antibacterial or antifungal agents with a new mechanism of action. Overall, this field, still in its infancy, may offer exciting new developments in the field of CAIs with a variety of pharmacological activities.

## Figures and Tables

**Table 1:** CA inhibition profile for Psammaplin A **1** and the reference CAI acetazolamide **AZA**.

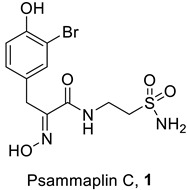		
**hCA Isoform**	**K_I_ (nM)**	
	**1**	**AZA**
hCA I	48.1	250
hCA II	88.0	12
hCA IV	75.3	74.2
hCA VA	154	63.1
hCA VI	9680	11.0
hCA VII	1.7	2.5
hCA IX	12.3	25.0
hCA XII	0.79	5.7
hCA XIII	10630	16.4
hCA XIV	379	41.3

**Table 2:** hCA inhibition data with marine NP polyamines **28**–**32**. Inhibition data with spermine and spermidine [47] as standard polyamines as well as with the clinically used sulfonamide CAI acetazolamide (AAZ) are also shown.

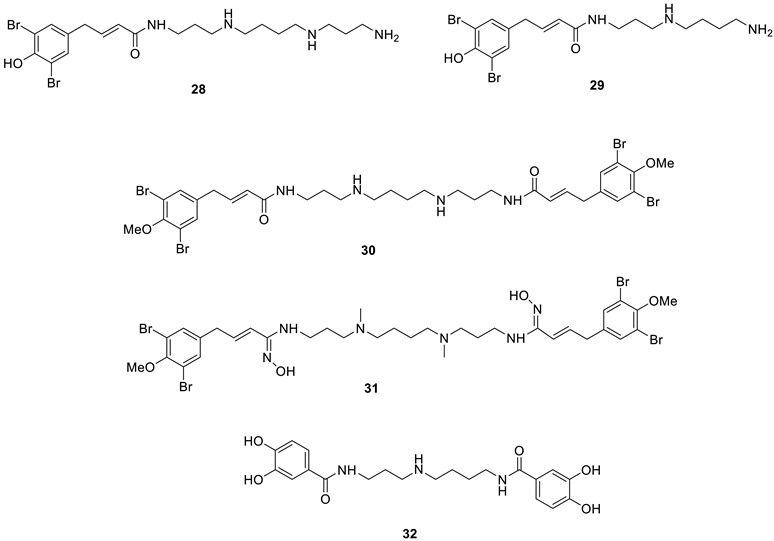						
**Polyamine**	**K_I_ (** **μM)**					
	**hCA I**	**hCA II**	**hCA IV**	**hCA IX**	**hCA XII**	**hCA XIV**
Spermine	231	84	0.010	13.3	27.6	0.86
Spermidine	1.40	1.11	0.112	1.37	44.1	1.00
**28**	1.76	0.41	6.72	0.20	2.81	2.12
**29**	0.77	0.37	9.10	0.35	3.48	2.28
**30**	0.86	0.35	9.08	0.27	3.50	6.96
**31**	0.85	0.48	>20	0.34	>20	2.72
**32**	0.79	0.34	7.03	0.36	4.21	1.52
AAZ	0.25	0.012	0.074	0.025	0.006	0.041

**Table 3:** Ascidian-derived NP coumarins **37**–**39** and their CA inhibitory properties.

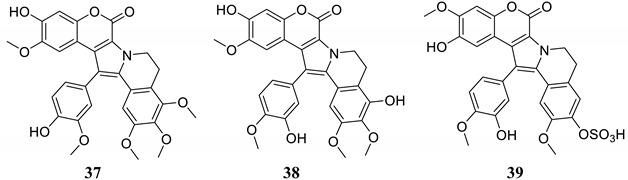						
**Compound**	**K_I_ (μM)**					
	**hCA I**	**hCA II**	**hCA VII**	**hCA IX**	**hCA XII**	**hCA XIII**
**37**	6.45	>100	14.5	3.22	9.07	4.63
**38**	6.55	>100	78.4	3.27	1.79	4.24
**39**	40.1	>100	58.3	6.33	8.51	3.70

**Table 4:** CA inhibition data of enzymes from pathogenic bacteria (*Mycobaterium tuberculosis* β-CA isozymes Rv3273 and Rv1284), fungi (*Candida albicans* isozyme Nce103 and *Cryptococcus neoformans* isozyme Can2) and human α-CA isozymes I and II, with the NP-based compounds polyandrocarpamine A (**40**) and polyandrocarpamine B (**41**), the standard sulfonamide CA inhibitor, **AZA**, and phenol.

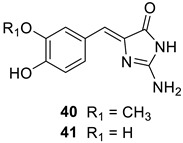						
**Compound**	**K_I_ (** **μM)**					
	**Rv3273**	**Rv1284**	**Nce103**	**Can2**	**hCA I**	**hCA II**
**40**	0.91	11.8	0.92	0.89	10.5	9.6
**41**	0.92	0.91	0.90	0.95	355	13.1
**Phenol**	79.0	64.0	17.3	25.9	10.1	5.5
**AZA**	0.10	0.48	0.13	0.01	0.25	0.012
